# Supplementary material for: Effects of different exercise types on vascular endothelial function in individuals with abnormal glycaemic control: a systematic review and network meta-analysis
Source: PeerJ. 2025 Aug 8;13:e19839. doi: 10.7717/peerj.19839 (PMC12338059; doi:10.7717/peerj.19839)

| **Supplemental materials** | **Page** |
| --- | --- |
| **Appendix S1.** Search strategy of PubMed, Embase, Cochrane, Web of Science and EBSCO. | 2-4 |
| **Appendix S2.** Sensitivity analysis of pooled effect estimates under different correlation coefficients. | 5 |
| **Appendix S3.** Characteristics of the included studies. | 6-9 |
| **Appendix S4.** Risk of bias assessment. | 10 |
| **Appendix S5.** Contributions of direct and indirect comparisons to NMA and the number of studies of each direct comparison of brachial artery flow-mediated dilation. | 11 |
| **Appendix S6.** Inconsistency of brachial artery flow-mediated dilation outcome tested by loop-specific heterogeneity estimates, inconsistency model, and node splitting analysis. | 12 |
| **Appendix S7.** Forest plots of eligible comparisons of brachial artery flow mediated dilation. | 13 |
| **Appendix S8.** Area under the curve for cumulative ranking probability of each intervention on brachial artery flow-mediated dilation. | 14 |
| **Appendix S9.** The funnel plot graphics of brachial artery flow-mediated dilation. | 15 |

**Appendix S1.** Search strategy of PubMed, Cochrane, Embase, Web of Science and EBSCO.

| **Database** | **Search Terms** | |  |  |
| --- | --- | --- | --- | --- |
|  | **Exercise** | **Type 2 diabetes mellitus** | **Endothelial Function** | **Randomized controlled**  **trial** |
| PubMed [Title/Abstract] | Exercise [MeSH Terms] OR exercise OR physical activity OR physical exercise OR sports OR training OR aerobic training OR high intensity interval training OR interval training OR endurance training OR sprint interval training OR resistance training OR strength training OR combined training OR concurrent training OR resistance exercise OR aerobic exercise OR strength exercise OR exercise, isometric OR combined exercise OR Tai Chi OR Taijiquan OR Baduanjin OR Yijinjing OR Wuqinxi OR Qigong OR Swimming OR whole body vibration training OR whole body vibration exercise OR whole body vibration therapy OR vibration Training OR vibration exercise OR oscillatory whole body stimulation | Diabetes mellitus, type 2 [MeSH Terms] OR type 2 diabetes mellitus OR type 2 diabetes OR diabetes, type 2 OR T2DM OR non-insulin-dependent diabetes mellitus OR diabetes mellitus, noninsulin-dependent OR NIDDM OR adult-onset diabetes OR diabetes mellitus, ketosis resistant OR diabetes mellitus, stable OR maturity onset diabetes mellitus OR MODY OR maturity-onset diabetes OR diabetes mellitus, adult onset OR prediabetes OR impaired glucose tolerance OR insulin resistance OR impaired fasting glucose OR adiponectin OR leptin OR adipokines OR glucose | Endothelial function OR endothelial OR endothelium OR vascular endothelium OR endothelial cells OR flow mediated dilation OR flow-mediated dilation OR FMD | Randomized controlled trial OR randomized OR placebo |
| Embase [Title/Abstract] | Training [Emtree term] OR exercise OR physical activity OR physical exercise OR sports OR training OR aerobic training OR high intensity interval training OR interval training OR endurance training OR sprint interval training OR resistance training OR strength training OR combined training OR concurrent training OR resistance exercise OR aerobic exercise OR strength exercise OR exercise, isometric OR combined exercise OR Tai Chi OR Taijiquan OR Baduanjin OR Yijinjing OR Wuqinxi OR Qigong OR Swimming OR whole body vibration training OR whole body vibration exercise OR whole body vibration therapy OR vibration Training OR Vibration Exercise OR oscillatory whole body stimulation | Type 2 diabetes mellitus [Emtree term] OR type 2 diabetes mellitus OR type 2 diabetes OR diabetes, type 2 OR T2DM OR non-insulin-dependent diabetes mellitus OR diabetes mellitus, noninsulin-dependent OR NIDDM OR adult-onset diabetes OR diabetes mellitus, ketosis resistant OR diabetes mellitus, stable OR maturity onset diabetes mellitus OR MODY OR maturity-onset diabetes OR diabetes mellitus, adult onset OR prediabetes OR impaired glucose tolerance OR insulin resistance OR impaired fasting glucose OR adiponectin OR leptin OR adipokines OR glucose | Endothelial function OR endothelial OR endothelium OR vascular endothelium OR endothelial cells OR flow mediated dilation OR flow-mediated dilation OR FMD | Randomized controlled trial OR randomized OR placebo |
| Cochrane [Title/Abstract/ keywords] | Exercise [MeSH Terms] OR exercise OR physical activity OR physical exercise OR sports OR training OR aerobic training OR high intensity interval training OR interval training OR endurance training OR sprint interval training OR resistance training OR strength training OR combined training OR concurrent training OR resistance exercise OR aerobic exercise OR strength exercise OR exercise, isometric OR combined exercise OR Tai Chi OR Taijiquan OR Baduanjin OR Yijinjing OR Wuqinxi OR Qigong OR Swimming OR whole body vibration training OR whole body vibration exercise OR whole body vibration therapy OR vibration Training OR Vibration Exercise OR oscillatory whole body stimulation | Diabetes mellitus, type 2 [MeSH Terms] OR type 2 diabetes mellitus OR type 2 diabetes OR diabetes, type 2 OR T2DM OR non-insulin-dependent diabetes mellitus OR diabetes mellitus, noninsulin-dependent OR NIDDM OR adult-onset diabetes OR diabetes mellitus, ketosis resistant OR diabetes mellitus, stable OR maturity onset diabetes mellitus OR MODY OR maturity-onset diabetes OR diabetes mellitus, adult onset OR prediabetes OR impaired glucose tolerance OR insulin resistance OR impaired fasting glucose OR adiponectin OR leptin OR adipokines OR glucose | Endothelial function OR endothelial OR endothelium OR vascular endothelium OR endothelial cells OR flow mediated dilation OR flow-mediated dilation OR FMD | — |
| Web of Science  [Topic] | TS=Exercise OR TS=physical activity OR TS=physical exercise OR TS=sports OR TS=training OR TS=aerobic training OR TS=high intensity interval training OR TS=interval training OR TS=endurance training OR TS=sprint interval training OR TS=resistance training OR TS=strength training OR TS=combined training OR TS=concurrent training OR TS=resistance exercise OR TS=aerobic exercise OR TS=strength exercise OR TS=exercise, isometric OR TS=combined exercise OR TS=Tai Chi OR TS=Taijiquan OR TS=Baduanjin OR TS=Yijinjing OR TS=Wuqinxi OR TS=Qigong OR TS=Swimming OR TS=whole body vibration training OR TS=whole body vibration exercise OR TS=whole body vibration therapy OR TS=vibration Training OR TS=vibration Exercise OR TS=oscillatory whole body stimulation | TS=Diabetes mellitus, type 2 OR TS=type 2 diabetes mellitus OR TS=type 2 diabetes OR TS=diabetes, type 2 OR TS=T2DM OR TS=non-insulin-dependent diabetes mellitus OR TS=diabetes mellitus, noninsulin-dependent OR TS=NIDDM OR TS=adult-onset diabetes OR TS=diabetes mellitus, ketosis resistant OR TS=diabetes mellitus, stable OR TS=maturity onset diabetes mellitus OR TS=MODY OR TS=maturity-onset diabetes OR TS=diabetes mellitus, adult onset OR TS=prediabetes OR TS=impaired glucose tolerance OR TS=insulin resistance OR TS=impaired fasting glucose OR TS=adiponectin OR TS=leptin OR TS=adipokines OR TS=glucose | TS=Endothelial function OR TS=endothelial OR TS=endothelium OR TS=vascular endothelium OR TS=endothelial cells OR TS=flow mediated dilation OR TS=flow-mediated dilation OR TS=FMD | TS=Randomized controlled trial OR TS=randomized OR TS=placebo |
| EBSCO  [Abstract] | Exercise OR physical activity OR physical exercise OR sports OR training OR aerobic training OR high intensity interval training OR interval training OR endurance training OR sprint interval training OR resistance training OR strength training OR combined training OR concurrent training OR resistance exercise OR aerobic exercise OR strength exercise OR exercise, isometric OR combined exercise OR Tai Chi OR Taijiquan OR Baduanjin OR Yijinjing OR Wuqinxi OR Qigong OR Swimming OR whole body vibration training OR whole body vibration exercise OR whole body vibration therapy OR vibration Training OR Vibration Exercise OR oscillatory whole body stimulation | Diabetes mellitus, type 2 OR type 2 diabetes mellitus OR type 2 diabetes OR diabetes, type 2 OR T2DM OR non-insulin-dependent diabetes mellitus OR diabetes mellitus, noninsulin-dependent OR NIDDM OR adult-onset diabetes OR diabetes mellitus, ketosis resistant OR diabetes mellitus, stable OR maturity onset diabetes mellitus OR MODY OR maturity-onset diabetes OR diabetes mellitus, adult onset OR prediabetes OR impaired glucose tolerance OR insulin resistance OR impaired fasting glucose OR adiponectin OR leptin OR adipokines OR glucose | Endothelial function OR endothelial OR endothelium OR vascular endothelium OR endothelial cells OR flow mediated dilation OR flow-mediated dilation OR FMD | Randomized controlled trial OR randomized OR placebo |

**Appendix S2.** Sensitivity analysis of pooled effect estimates under different correlation coefficients (Corr).

| Corr | AIE *vs.* CON | MBE *vs.* CON | CE *vs.* CON | ACE *vs.* CON | RE *vs.* CON | Ranking (SUCRA) (1st/2nd/3rd) |
| --- | --- | --- | --- | --- | --- | --- |
| 0.3 | 2.21 (1.05, 3.37) | 1.94 (0.58, 3.31) | 1.12 (0.11, 2.12) | 1.10 (0.40, 1.80) | 0.08 (-2.03, 2.18) | AIE (89.6%) > MBE (80.2) > CE (51.0) |
| 0.5 | 2.23 (1.09, 3.37) | 1.97 (0.60, 3.33) | 1.17 (0.13, 2.21) | 1.20 (0.52, 1.87) | 0.18 (-1.78, 2.15) | AIE (89.0%) > MBE (80.1) > CE (51.0) |
| 0.7 | 2.27 (1.13, 3.41) | 2.00 (0.60, 3.40) | 1.25 (0.17, 2.33) | 1.32 (0.65, 1.98) | 0.30 (-1.54, 2.14) | AIE (88.3%) > MBE (78.7) > ACE (52.8) |

Note:Effect Measure: [ Mean Difference (MD), 95% CrI]; CON, control; ACE, aerobic continuous exercise; AIE, aerobic interval exercise; RE, resistance exercise; CE, combined exercise; MBE, mind-body exercise.

Sensitivity analyses showed consistent rankings (AIE as the best intervention) and effect estimates across all Corr values (0.3–0.7), with overlapping credible intervals (**Appendix S2**).

**Appendix S3.** Characteristics of the included studies.

| study | Population | Sample size | %  Female | Mean  age | Exercise type | Summary description of exercise intervention (frequency, intensity, time excluding warm-up/cool-down and type) | Duration (wks) | supervised or nonsupervised |
| --- | --- | --- | --- | --- | --- | --- | --- | --- |
| Abdi et al. (2021) | T2DM | 15 | 100.0 | 33.0 | CON | No exercise | 12 | supervised |
|  |  | 15 | 100.0 | 33.0 | AIE | 3 d/wek; 4 sets* 4 min at 85%-95% HRmax; active rest intervals: 3 min at 50-60% HRmax; treadmill training | 12 | supervised |
| Afousi et al. (2018) | T2DM | 17 | 47.1 | 54.2 | CON | No exercise | 12 | supervised |
|  |  | 17 | 58.8 | 53.1 | ACE | 3 d/wek; 70% HRmax; 42 min; cycling | 12 | supervised |
|  |  | 18 | 50.0 | 54.8 | AIE | 3 d/wek; 12 sets* 1.5 min at 85%-90% HRmax; active rest intervals: 2 min at 55-60% HRmax; cycling | 12 | supervised |
| Choi et al.  (2012) | T2DM | 37 | 100.0 | 55.0 | CON | No exercise | 12 | supervised |
|  |  | 38 | 100.0 | 53.8 | ACE | 5 d/wek; moderate intensity; 60 min; walking | 12 | supervised |
| Cox et al.  ( 2024) | T2DM | 23 | 39.1 | 59.4 | CON | No exercise | 8 | supervised |
|  |  | 23 | 39.1 | 59.0 | CE | 3 d/wek; ACE (4 min 85%–95% HRmax; treadmill training, cycling) and RE (8 sets* 1 min at RPE≧ 17; machine-based, bodyweight, and free-weight exercises) total: 26min | 8 | supervised |
| Davoodi et al. (2022) | T2DM | 15 | 46.7 | 53.7 | CON | No exercise | 12 | supervised |
|  |  | 16 | 37.5 | 54.9 | ACE | 3 d/wek; 70% HRmax; 42 min; cycling | 12 | supervised |
|  |  | 16 | 43.8 | 52.5 | AIE | 3 d/wek; 12 sets* 1.5 min at 85%-90% HRmax; active rest intervals: 2 min at 55-60% HRmax; cycling | 12 | supervised |
| Desch et al.  (2010) | Prediabetes | 12 | 34.0 | 62.3 | CON | No exercise | 24 | supervised |
|  |  | 14 | 22.0 | 62.3 | ACE | 2 d/wek; 75% HRmax; 90 min; cycling | 24 | supervised |
| Gainey et al. (2016) | T2DM | 11 | 81.8 | 63 | ACE | 3 d/wek; 1-6 weeks: 50%-60% HRmax; 30 min; 7-12 weeks: 60%-70% HRmax; 30 min; traditional walking | 12 | supervised |
|  |  | 12 | 83.3 | 58 | MBE | 3 d/wek; 1-6 weeks: 50%-60% HRmax; 30 min; 7-12 weeks: 60%-70% HRmax; 30 min; walking meditation | 12 | supervised |
| Gibbs et al.  (2012) | T2DM | 63 | 41.0 | 56 | CON | No exercise | 24 | supervised |
|  |  | 49 | 34.0 | 58 | CE | 3 d/wek; ACE (60%-90% HRmax; 45 min) and RE (2* 12-15 at 50%1RM;latisissimus dorsi pull down, leg extension, leg curl, bench press, leg press, shoulder press, and seated mid-rowing) | 24 | supervised |
| Ku et al.  (2009) | T2DM | 18 | 100.0 | 57.3 | CON | No exercise | 12 | supervised |
|  |  | 17 | 100.0 | 55.7 | ACE | 5 d/wek; 3.6-5.2 METs; 60 min; walking | 12 | supervised |
| Kwon et al.  (2011) | T2DM | 15 | 100.0 | 58.9 | CON | No exercise | 12 | supervised |
|  |  | 13 | 100.0 | 55.5 | ACE | 5 d/wek; 3.6-6.0 METs; 60 min; walking | 12 | supervised |
|  |  | 12 | 100.0 | 56.3 | RE | 3 d/wek; 3 sets* 10-15; 40min; resistance bands; bicep  curls, tricep extensions, upright rows, shoulder chest press, seated rows, trunk side bends, leg press, hip flexions, leg flexions, and leg extensions | 12 | supervised |
| Li  (2009) | T2DM | 39 | 35.9 | 56.5 | CON | No exercise | 24 | supervised |
|  |  | 40 | 30.0 | 57.8 | MBE | 5 d/wek; HR=(170-180)-age beats/min; 60min; Baduanjin | 24 | supervised |
| Liu et al.  (2007) | Prediabetes | 17 | NR | 49.8 | CON | No exercise | 24 | supervised |
|  |  | 17 | NR | 49.8 | ACE | 4 d/wek, 60%-70% HRmax; 50 min; walking | 24 | supervised |
|  |  | 16 | NR | 49.8 | CE | 4 d/wek, ACE (60%-70% HRmax; 20 min; walking) and RE (2-3sets* 15-20; 30min; Lat pulldown, straight-arm front raise, sit-up, supine straight-leg raise, leg press and leg extension) | 24 | supervised |
| Mitranun et al. (2014) | T2DM | 15 | 66.7 | 60.9 | CON | No exercise | 12 | supervised |
|  |  | 14 | 64.3 | 61.7 | ACE | 3 d/wek; 1-2 weeks: 50% VO_2_peak; 20min; treadmill training; 3-6 weeks: 60% VO_2_peak; 20min; treadmill training; 7-12 weeks: 65% VO_2_peak; 30min; treadmill training | 12 | supervised |
|  |  | 14 | 64.3 | 61.2 | AIE | 3 d/wek; 1-2 weeks: 50% VO_2_peak; 20min; treadmill training; 3-6 weeks: 4 sets* 1min 80% VO_2_peak; active rest intervals: 4 min 50% VO_2_peak; treadmill training; 7-12 weeks: 6 sets* 1min 85% VO_2_peak; active rest intervals: 4 min 60% VO_2_peak; treadmill training | 12 | supervised |
| Okada et al.  (2010) | T2DM | 17 | 35.3 | 64.5 | CON | No exercise | 12 | supervised |
|  |  | 21 | 52.3 | 61.9 | CE | 3-5 d/wek; ACE (20 min aerobic dance and 20 min stationary bicycle riding) and RE (20 min resistance training) | 12 | supervised |
| Ploydang et al. (2023) | T2DM | 17 | 58.8 | 69.2 | CON | No exercise | 12 | supervised |
|  |  | 16 | 68.8 | 68.9 | ACE | 3 d/wek; 1-6 weeks: 40%-50% HRR; 40min; nordic walking in water; 7-12 weeks: 50%-60% HRR; 40min; nordic walking in water | 12 | supervised |
| Rech et al. (2019) | T2DM | 21 | 52.4 | 68.0 | CON | Slight stretching | 12 | supervised |
|  |  | 17 | 41.2 | 70.5 | RE | 3 d/wek; 1-4 weeks: 2 sets* 10-12; 5-8 weeks: 3 sets* 10-12; 9-12 weeks: 3 sets* 10-15; unilateral leg press, unilateral knee extension, knee flexion, plantar flexion, bench press, low row, biceps curl, elbow extension, hip abduction, abdominal crunches, squat and bench stepping | 12 | supervised |
| Wycherley et al. (2008) | T2DM | 16 | 37.5 | 53.0 | CON | No exercise | 12 | supervised |
|  |  | 13 | 53.8 | 51.7 | ACE | 4-5 d/wek; 1-3 weeks: 60%-70%HRmax; 25-40min; 4-7 weesk: 65%-75%HRmax; 35-55min; 8-12 weeks: 70%-80%HRmax; 50-60min; walking/jogging | 12 | supervised |

Abbreviations: CON, control; ACE, aerobic continuous exercise; AIE, aerobic interval exercise; RE, resistance exercise; CE, combined exercise; MBE, mind-body exercise; RM, repetition maximum; RPE, rating of perceived exertion; VO_2_peak, peak oxygen uptake; HRmax, maximum heart rate; HRR, heart rate reserve.

**Appendix S4.** Risk of bias assessment.


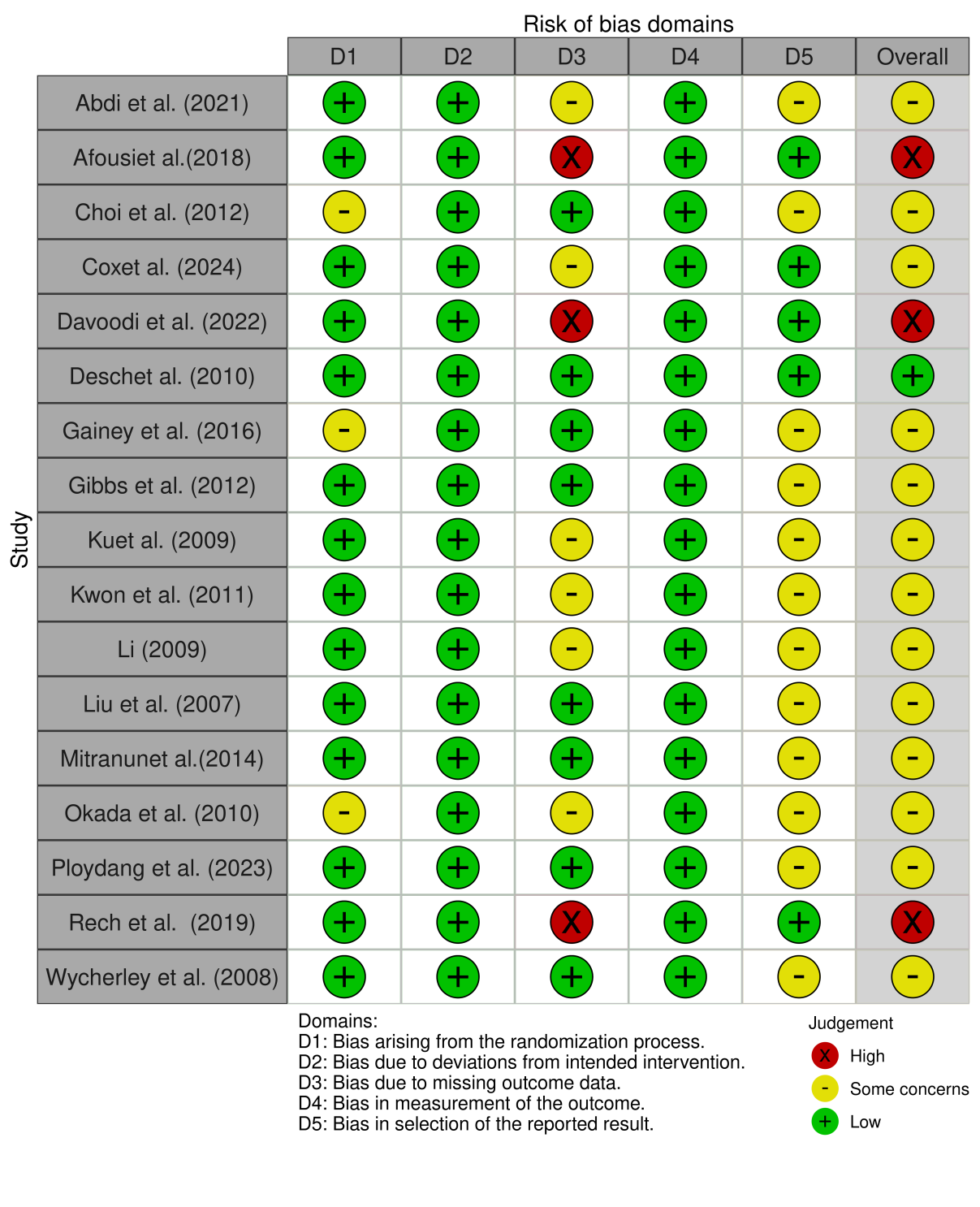


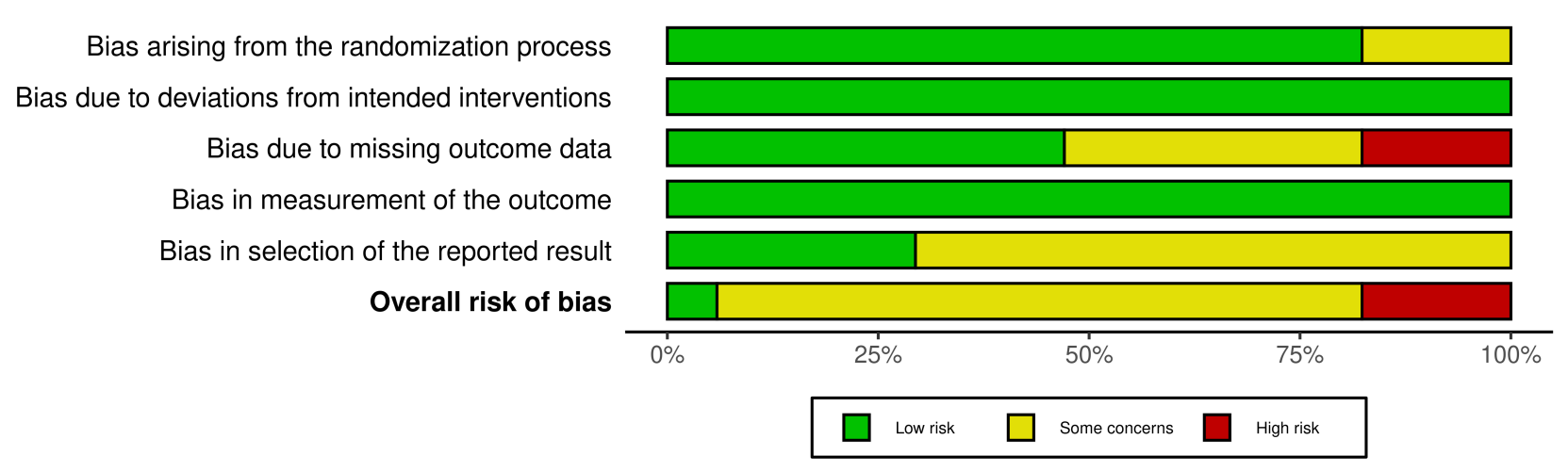


**Appendix S5.** Contributions of direct and indirect comparisons to NMA and the number of studies of each direct comparison of brachial artery flow-mediated dilation.

**
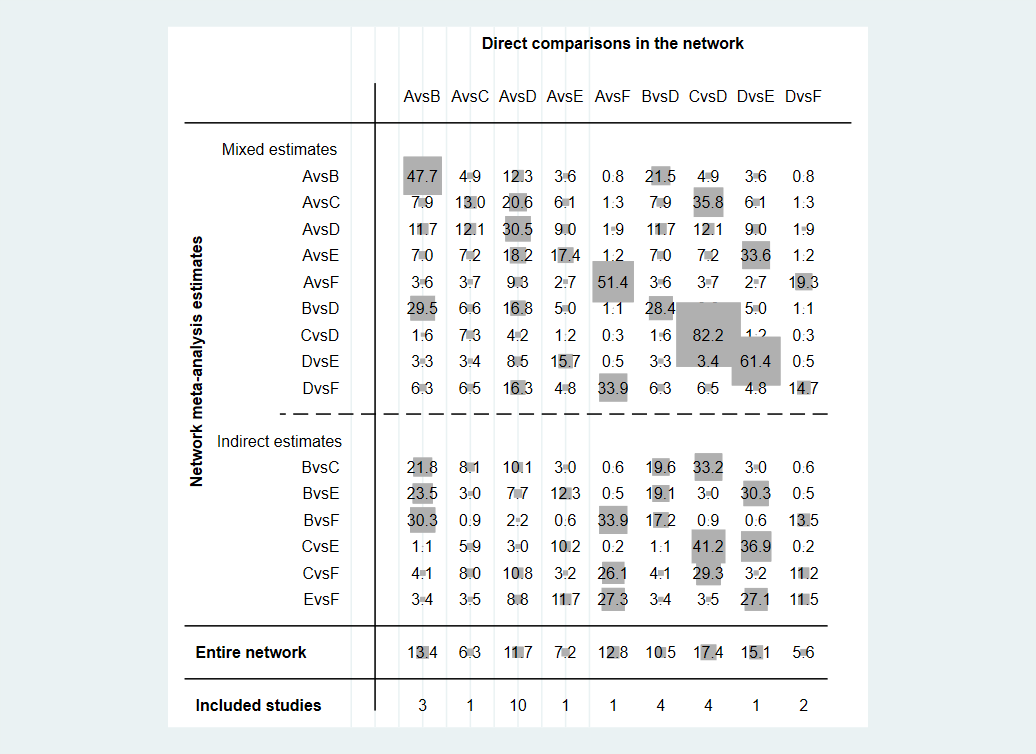
**

Note: A: ACE, B: AIE, C: CE, D: CON, E: MBE, F: RE.

**Appendix S6.** Inconsistency of brachial artery flow-mediated dilation outcome tested by loop-specific heterogeneity estimates, inconsistency model, and node splitting analysis.

**loop-specific heterogeneity estimates**

| **Loop** | **IF** | **seIF** | **z_value** | **p_value** | **CI_95** | **Loop_Hete rog_tau2** |
| --- | --- | --- | --- | --- | --- | --- |
| A-D-F | 1.082 | 1.886 | 0.574 | 0.566 | (0.00,4.78) | 0.930 |
| A-D-E | 0.776 | 1.818 | 0.421 | 0.673 | (0.00,4.33) | 1.242 |
| A-C-D | 0.427 | 1.468 | 0.291 | 0.771 | (0.00,3.30) | 1.104 |
| A-B-D | 0.044 | 1.261 | 0.035 | 0.972 | (0.00,2.52) | 1.377 |

Note: A: ACE, B: AIE, C: CE, D: CON, E: MBE, F: RE.

**Inconsistency model**

| chi2 (7) | 6.01 |
| --- | --- |
| Prob > chi2 | 0.5387 |

**Node splitting analysis**

| **Side** | **Direct** |  | **Indirect** |  | **Difference** |  |  |  |
| --- | --- | --- | --- | --- | --- | --- | --- | --- |
|  | **Coef.** | **Std. Err.** | **Coef.** | **Std. Err.** | **Coef.** | **Std. Err.** | **P>\|z\|** | **tau** |
| A B | 0.98 | 0.66 | 1.26 | 1.26 | -0.28 | 1.40 | 0.84 | 0.84 |
| A C | 0.31 | 1.07 | -0.21 | 0.75 | 0.53 | 1.30 | 0.69 | 0.85 |
| A D | -1.15 | 0.37 | -1.81 | 1.15 | 0.66 | 1.19 | 0.58 | 0.83 |
| A E | 0.30 | 1.08 | 1.14 | 0.98 | -0.84 | 1.46 | 0.56 | 0.83 |
| A F | -1.59 | 1.17 | 0.18 | 1.65 | -1.77 | 1.92 | 0.36 | 0.74 |
| B D * | -2.20 | 0.64 | -2.56 | 1.42 | 0.35 | 1.51 | 0.82 | 0.85 |
| C D * | -1.12 | 0.56 | -2.08 | 1.91 | 0.96 | 1.94 | 0.62 | 0.86 |
| D E | 2.30 | 0.91 | 1.46 | 1.14 | 0.84 | 1.46 | 0.56 | 0.83 |
| D F ***** | 0.79 | 1.03 | -2.38 | 1.93 | 3.17 | 2.07 | 0.13 | 0.69 |
| **Note:** A: ACE, B: AIE, C: CE, D: CON, E: MBE, F: RE. | | | | | | | |  |

* All the evidence about these contrasts comes from the trials which directly compare them.

**Appendix S7.** Forest plots of eligible comparisons of brachial artery flow-mediated dilation.


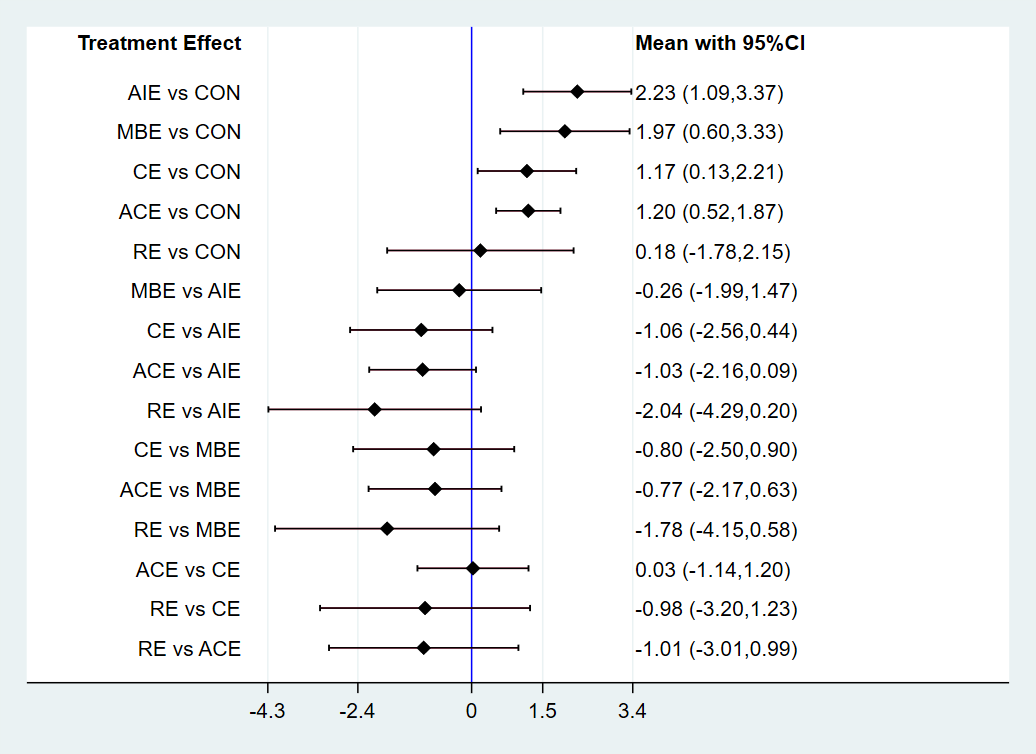


**Appendix S8.** Area under the curve for cumulative ranking probability of each intervention on brachial artery flow-mediated dilation.


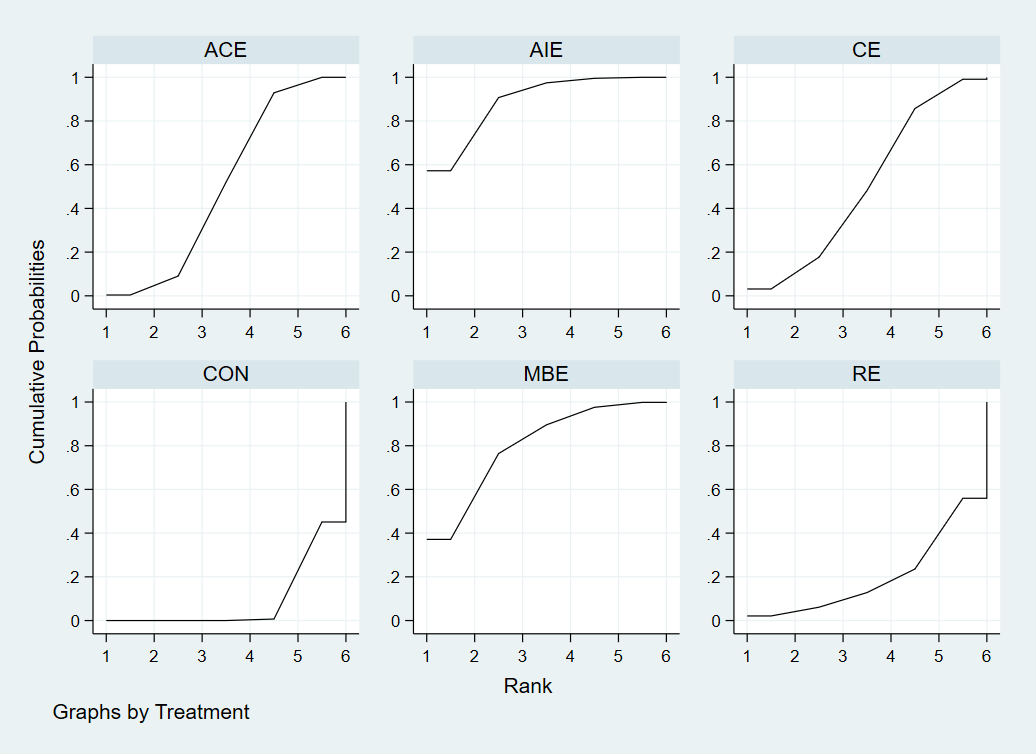


**Appendix S9.** The funnel plot graphics of brachial artery flow-mediated dilation.


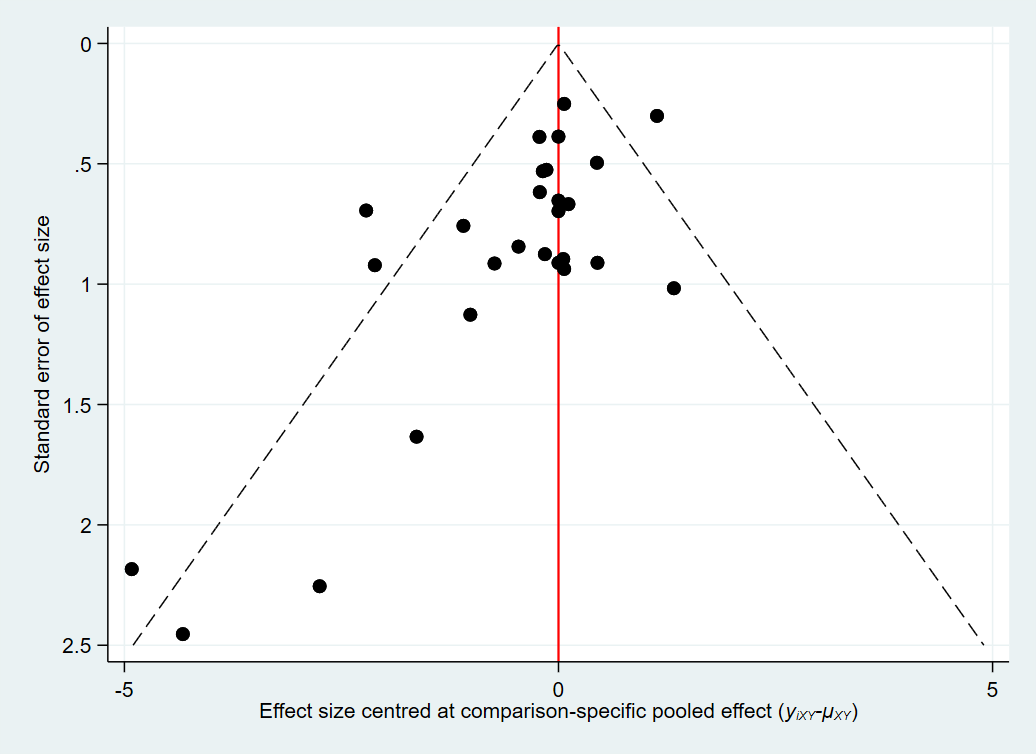

Supplement: Supplemental Information 2 [file peerj-13-19839-s002.docx]
